# PAK1 inhibitor NVS‐PAK1‐1 inhibits degeneration of synaptic spines in cultured neurons exposed to amyloid or tau and in 5XFAD mice following oral administration

**DOI:** 10.1002/alz70859_103176

**Published:** 2025-12-25

**Authors:** Frank Longo

**Affiliations:** ^1^ Stanford University, Stanford, CA USA

## Abstract

**Background:**

In Alzheimer’s disease (AD), degeneration of synapses, including synaptic spines, is highly correlated with loss of cognition. p21‐activated kinase 1 (PAK1) modulates cofilin, an actin‐binding protein regulating spine integrity. PAK1 was prioritized as a therapeutic target based on genome‐wide analysis of genetic and multi‐omics data, with protein abundance changes validated in multiple AD mouse models. Concomitant with cognitive loss, PAK1 is translocated to the cell membrane, potentially disrupting regulation of cofilin. We tested the hypothesis that inhibition of PAK1 would decrease loss of synaptic spines in the 5XFAD model.

**Method:**

In in vitro studies, oligomeric amyloid‐beta (Aβ) or tau was applied to matured mouse hippocampal neurons with or without NVS‐PAK1‐1, a selective allosteric PAK1 inhibitor, and dendritic spines were quantitated. NVS‐PAK1‐1 was orally administered to CD‐1 and 5XFAD mice for PK‐PD analysis and quantitation of dendritic spines using Golgi staining. PAK1 activity was assessed using western blotting for pPAK1.

**Result:**

In culture studies, NVS‐PAK1‐1 but not its analog negative control, resulted in resilience of spines to Aβ and tau oligomers, with an EC50 of 1 nM. CD‐1 mouse studies demonstrated oral bioavailability, and brain levels well above the in vitro EC50 following peripheral administration, In 5XFAD mice, oral administration of NVS‐PAK1 demonstrated dose‐ and time‐dependent drug levels in plasma and brain, and dose‐dependent inhibition of PAK1 activity (pPAK1 levels), confirming in vivo target engagement. Administration of NVS‐PAK1‐1 (100 mg/kg, PO, BID) to 3‐month old 5XFAD mice, at onset of amyloid pathology, and continuing until 6 months of age, resulted in reduced pPAK1 levels. There was no significant attenuation of amyloid levels. Quantitative analysis of somatosensory dendritic spine density demonstrated reduced density in vehicle‐treated 5XFAD compared to wild type mice. Administration of NVS‐PAK1‐1 to 5XFAD mice resulted in spine density matching that of age‐ and sex‐matched vehicle treated WT littermate wild type mice.

**Conclusion:**

These data support the hypothesis that inhibition of PAK1 confers dendritic spine resilience against Aβ and tau oligomers in early‐stage preclinical models. Ongoing oncology clinical trials assessing effects of PAK1 inhibitors point to the possibility of testing PAK1 inhibitor approaches in AD trials.

